# Leveraging Clinical Decision Support in Dental Settings to Bridge HIV Testing Gaps and Contribute to Ending the Epidemic

**DOI:** 10.1055/a-2500-7594

**Published:** 2025-02-05

**Authors:** Sharon C. Perelman, Tunaidi Ansari, Michael T. Yin, Peter G. Gordon, Nadia Nguyen, Vicky Evangelidis-Sakellson, Carol Kunzel, Kathrine Meyers, Delivette Castor, Ariel Blanchard, David A. Albert

**Affiliations:** 1 Department of Operative Dentistry, Columbia University College of Dental Medicine, New York, New York, United States; 2 Columbia University College of Dental Medicine, New York, New York, United States; 3 Division of Infectious Diseases, Department of Medicine, Columbia University Irving Medical Center, New York, New York, United States; 4 Department of Foundational Sciences, Columbia University College of Dental Medicine, New York, New York, United States; 5 Division of Oral & Maxillofacial Surgery, Department of Surgery, New York-Presbyterian Hospital—Weill Cornell Medical Center, New York, New York, United States; 6 Department of Oral and Maxillofacial Surgery, Columbia University College of Dental Medicine, Mailman School of Public Health, New York, New York, United States

**Keywords:** electronic health records and systems, clinical decision support systems, human immunodeficiency virus, point-of-care diagnostic testing, dental clinic

## Abstract

**Background** Columbia University Irving Medical Center (CUIMC) in New York City, in collaboration with the Division of Infectious Diseases and the Dental School, is addressing a critical gap in HIV testing to support the strategy to End the HIV Epidemic (EHE). This strategy emphasizes increasing testing rates and providing patients with information about pre-exposure prophylaxis (PrEP).

**Objectives** This study aimed to achieve two key objectives: (1) develop a robust clinical decision support system (CDSS) capable of identifying patients who stand to benefit from HIV testing and (2) implement a seamlessly integrated, user-friendly workflow, enabling health care providers to effortlessly order and conduct HIV point-of-care (POC) screening.

**Methods** A targeted CDSS was developed by identifying a patient population, determining qualifying laboratory tests, interpreting HIV and sexually transmitted infections results, and programming based on conditional statements. A workflow was implemented after careful consideration and collaboration with faculty and residents. POC testing was conducted using the OraQuick Rapid Antibody Test Advanced HIV-1/2.

**Results** The implementation of this targeted CDSS and associated new protocols demonstrated a promising 11.5% testing rate, normalizing HIV POC testing within the dental ambulatory care setting, and representing a key pillar of EHE.

**Conclusion** CUIMC's approach presents a promising strategy for bridging gaps in HIV testing disparities and enhancing public health outcomes. By leveraging CDSS and innovative health care delivery methods, CUIMC's desire is to expand the scope and effectiveness of HIV testing to other practices and sites.

## Background and Significance

At the conclusion of 2022, the Centers for Disease Control and Prevention (CDC) estimated that 1.2 million individuals in the United States were living with HIV of which 156,000 remained unaware of their status. 1 Notably, 80% of new HIV infections are thought to stem from individuals who are unaware of their HIV-positive status or are not receiving care. 1 2

To reach the U.S.' Ending the HIV Epidemic (EHE) goals, according to The Joint United Nations Program on HIV/AIDS's (UNAIDS) fast-track targets, the health system must diagnose 95% of people with HIV as early as possible, provide immediate initiation or reinitiation of antiretroviral therapy in 95% of patients diagnosed and sustain antiretroviral therapy for HIV viral load suppression in 95% of those on treatment by 2030. 3

The standard of care is to offer initial HIV testing for individuals aged 13 and over and periodic retesting for those with HIV-related risk behaviors, including unprotected sex. 4 HIV and sexually transmitted infections (STI) such as syphilis, gonorrhea, and chlamydia are linked epidemiologically and by synergistic transmission. 5 6 Interventions like pre-exposure prophylaxis (PrEP) for HIV prevention, immediate antiretroviral therapy initiation, and programs to promote retention in care are recommended. 7 8

It is important to note that individuals diagnosed with HIV can lead healthy lives with antiretroviral therapy. 9 Additionally, those at high risk for HIV who test negative can reduce their risk of sexual transmission by 99% through the use of these highly effective antiretroviral medications when taken as prescribed. 9 In 2022, 36% of eligible individuals in the United States were prescribed PrEP; therefore, increasing this coverage is a crucial strategy in the EHE initiative. 10

People with HIV are particularly vulnerable to oral health issues. Common problems include chronic dry mouth, gum disease (gingivitis), bone loss around the teeth (periodontitis), canker sores, oral warts, fever blisters, thrush (oral candidiasis), hairy leukoplakia (which causes rough, white patches on the tongue), and tooth decay. 11

Expanding the workforce to health care providers within the dental clinics presents an untapped opportunity to EHE, especially since it is a site of contact for patients who may not be engaged in medical care. 2 4 12 13 Although HIV testing is well accepted in the dental clinic, there is concern regarding documented barriers to HIV testing by dental care providers. 14 15 16 17 These barriers include patient acceptability, gaps in knowledge needed to provide HIV testing and posttest counseling, time constraints during clinical encounters, resources, and sustainability. 14 15 16 17 18 19 20 21 Additionally, dentists may not feel comfortable addressing issues related to HIV risk and/or sexuality with patients. They require training on how to inform their patients about their HIV status and assist in establishing linkages with HIV-related medical services. 16 21 22 23

Approximately 70% of individuals who have never been tested for HIV have had contact with a dental provider. 15 In fact, a significant subset of patients who have not seen a general health care provider within a 2-year period have instead seen a dental provider. 24 The use of the electronic health record (EHR), Epic, has expanded into 1,800 dental practices and eight dental schools, reflecting the growing integration of medical and dental records. This unified platform allows clinical professionals to share data, enhancing treatment and public health initiatives. 25 Columbia University's College of Dental Medicine (CDM) is an integral part of Columbia University Irving Medical Center (CUIMC), located in New York City. This consortium operates on Epic, encompassing a diverse pool of over 4 million individual patient records. Throughout the fiscal years 2022 and 2023, the CDM dental clinics served over 60,000 unique patients. An analysis at CDM reveals that 63% of patients seen in the dental clinics have not utilized medical services within our consortium. Moreover, less than 3% of these patients had a documented HIV test in the EHR.

EHR reminders for HIV testing have demonstrated improvement in screening and/or testing for HIV. 26 27 28 Furthermore, clinical decision support systems (CDSS) have been shown to increase HIV testing and can be programmed to prompt retesting if an individual has ongoing HIV risk factors. 29 30 Like all CDSS tools, their effectiveness can be enhanced by incorporating the “five rights” for effective decision support: the right information to the right people, through the right channels, in the right intervention formats, and at the right points in the workflow. 31 32

In this paper, we will explain how our organization leveraged an untapped resource—dental clinics and dental providers—to expand HIV testing and developed a CDSS to accurately identify and target patients who would benefit most from HIV testing.

## Objectives

The Division of Infectious Diseases at CUIMC initiated a collaborative study with CDM to contribute to the broader goal of EHE. This study aims to develop a CDSS, referred to as a BestPractice Advisory within the EHR, designed to accurately pinpoint patients who would benefit from HIV testing. Additionally, we intend to introduce a streamlined and intuitive workflow for health care providers, facilitating seamless ordering, and execution of HIV point-of-care (POC) screening.

## Methods

### Building the Clinical Decision Support System

The first objective, developing the CDSS, involved identifying the target population for HIV testing. In this study, we included patients who are 18 years old or older with either (1) no history of HIV testing ever documented in the EHR or (2) a negative HIV test and a positive STI test within the past 2 years. While age is a simple data field, determining which HIV and STI tests to evaluate as well as their positive or negative status required significant clinical input, data analysis, data cleansing, and formulaic categorization of laboratory results.

The infectious diseases clinicians identified three STI's—chlamydia, gonorrhea, and syphilis—to consider. Subsequently, they determined which particular HIV and STI tests and their associated Logical Observation Identifiers Names and Codes (LOINC) to include in the evaluation ( Table 1 ).

**Table 1:** Reference table for HIV and sexually transmitted infection Logical Observation Identifiers Names and Codes utilized within the clinical decision support system development

LOINC	Name
56888-1	HIV 1 + 2 Ab + HIV1 p24 Ag [presence] in serum or plasma by immunoassay
80387-4	HIV 1 + 2 Ab [presence] in serum, plasma, or blood by rapid immunoassay
75666-8	HIV 1 + 2 Ab and HIV1 p24 Ag [identifier] in serum, plasma, or blood by rapid immunoassay
29893-5	HIV 1 Ab [presence] in serum or plasma by immunoassay
30361-0	HIV 2 Ab [presence] in serum or plasma by immunoassay
68961-2	HIV 1 Ab [presence] in serum, plasma, or blood by rapid immunoassay
81641-3	HIV 2 Ab [presence] in serum, plasma, or blood by rapid immunoassay
31201-7	HIV 1 + 2 Ab [presence] in serum or plasma by immunoassay
48345-3	HIV 1 + O + 2 Ab [presence] in serum or plasma
7918-6	Cells.CD3 + CD4 + CD8+ (double-positive)/100 cells in blood
80203-3	HIV 1 and 2 Ab [identifier] in serum, plasma, or blood by rapid immunoassay
80387-4	HIV 1 + 2 Ab [presence] in serum, plasma, or blood by rapid immunoassay
20447-9	HIV 1 RNA [#/volume] (viral load) in serum or plasma by NAA with probe detection
25835-0	HIV 1 RNA [presence] in serum or plasma by NAA with probe detection
44871-2	HIV 1 proviral DNA [presence] in blood by NAA with probe detection
5017-9	HIV 1 RNA [presence] in blood by NAA with probe detection
70241-5	HIV 1 RNA [#/volume] (viral load) in plasma by probe and target amplification method detection limit = 20 copies/mL
35437-3	HIV 1 Ab [presence] in saliva (oral fluid) by immunoassay
24111-7	*Neisseria gonorrhoeae* DNA [presence] in specimen by NAA with probe detection
21613-5	*Chlamydia trachomatis* DNA [presence] in specimen by NAA with probe detection
88718-2	*Chlamydophila pneumoniae* DNA [presence] in nasopharynx by NAA with probe detection
43304-5	*Chlamydia trachomatis* rRNA [presence] in specimen by NAA with probe detection
6357-8	*Chlamydia trachomatis* DNA [presence] in urine by NAA with probe detection
57288-3	*Chlamydia trachomatis* rRNA [presence] in nasopharynx by NAA with probe detection
50387-0	*Chlamydia trachomatis* rRNA [presence] in cervix by NAA with probe detection
11084-1	Reagin Ab [titer] in serum
31147-2	Reagin Ab [titer] in serum by RPR

Abbreviations: LOINC, Logical Observation Identifiers Names and Codes; STI, sexually transmitted infection.

Normalizing data are a crucial step in data preprocessing and analysis, ensuring that all features in a dataset have the same scale. This involves both manual and programmatic cleansing of the data. 33 34 Upon querying the EHR database for results pertaining to these tests, we noticed that actual results were not simply “positive” or “negative.” Some were stored as discrete values; however, most were free-text, some with spelling errors and others representing ambiguous interpretations. The dataset was extracted, formatted, and sorted by HIV and STI tests, from the most to the least common, to facilitate detailed review. The clinicians carefully reviewed and categorized the results as positive or negative. The next step involved developing logic that would consistently label such results in the same way. For example, a result with a value of “react” was determined to be positive; this was shorthand for “reactive,” indicating a positive result. However, the opposite of a reactive value is nonreactive, indicating a negative result. To programmatically identify a positive result, logic needed to accommodate other types of common result values or misspellings. For this reason, string-searching values for “reactive” and assuming the test was positive would not be correct most of the time—we would miss potential values “react” or incorrectly flag “nonreactive” as positive.

**Fig. 1:**
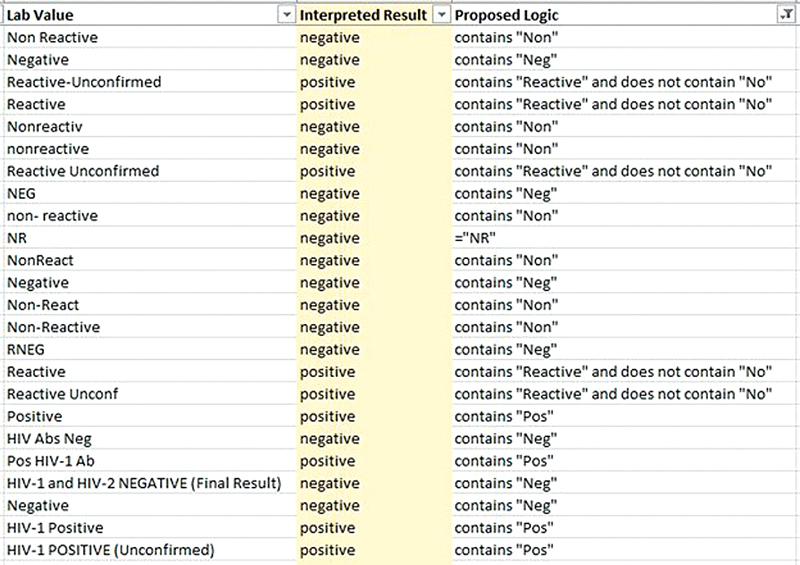
This figure reveals a small sample of the laboratory values within the electronic health record and the iterative work of interpreting the results with several combinations of logic.

The team went through many iterations, and any result with text displaying “neg” (considered to be “negative”) or “non” (considered to be “nonreactive”) or “nr” (also considered to be “nonreactive”) would be negative. On the contrary, any result with text displaying “pos” (considered to be “positive”), “react” but not containing “no” (considered to be “reactive”) or entered as detectable numerical values would be positive ( Fig. 1 ).

**Fig. 2:**
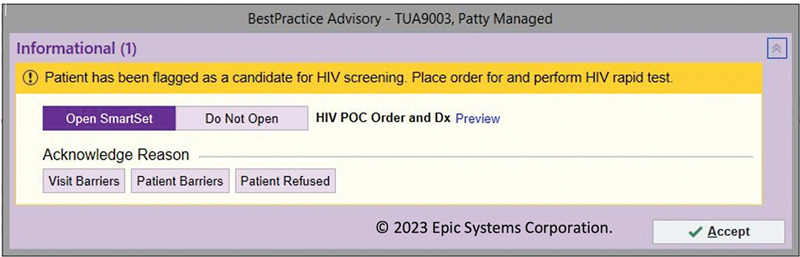
This figure shows the front-facing popup alert from the clinical decision support system within the Epic electronic health record.

Once finalized, we translated the logic into “rules,” which represents “if-then” conditional statements within the EHR's CDSS framework. Using “rules”-based programming, we ensured that the CDSS would be able to identify our target population and any exclusions; determined when and where the CDSS would fire; and configured the end user display, the permissible actions, and the recommended follow through ( Fig. 2 ).

### Developing the Workflow

The second objective in the study was to develop and implement a HIV POC testing workflow. We selected CDM's Advanced Education General Dentistry (AEGD) residency program to implement the workflow since these residents have already completed dental school and have experience treating a diverse patient population.

**Fig. 3:**
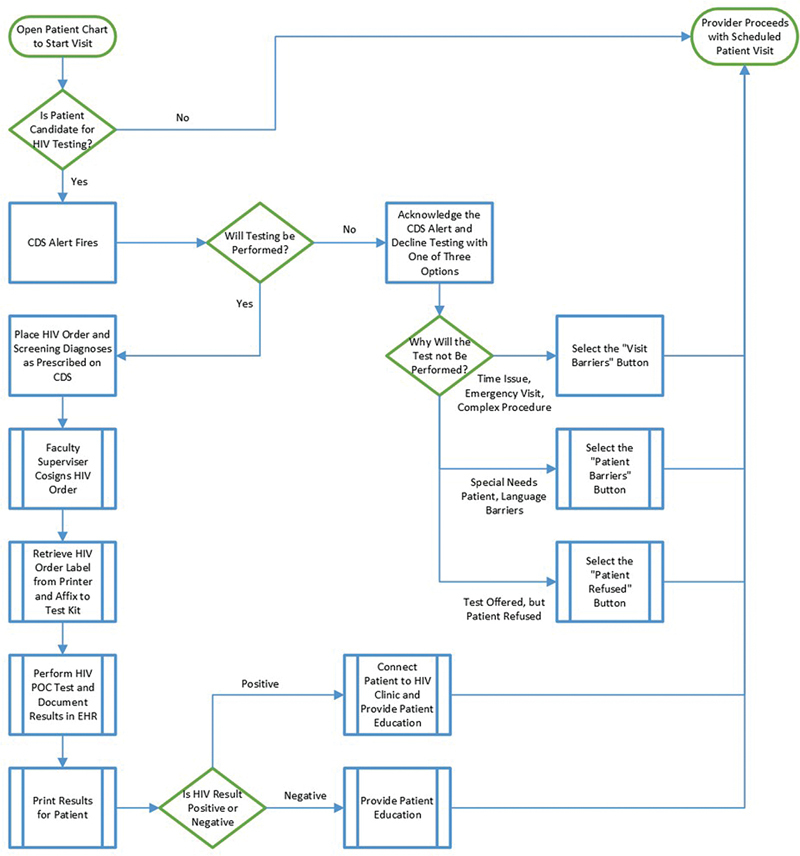
Diagram depicting end-to-end clinical workflow after clinical decision support system and HIV testing is implemented.

Collaborating with AEGD faculty, we determined operationally how best to design the workflow ( Fig. 3 ). The workflow in the AEGD clinic begins when the dental resident opens the patient's chart and the CDSS signals that the patient is a candidate for HIV testing. The resident offers HIV testing, and if the patient agrees, a preconfigured order set including the laboratory order and visit diagnosis, screening for HIV, is presented. If the test is declined, predefined reasons for not testing will display with subsequent delays in firing again depending on the selection.

The test utilized was the OraQuick Rapid Antibody Test Advanced HIV-1/2. It was chosen due to its ease of use, and although a comparison of rapid POC tests found that sensitivity of oral tests was slightly lower (98.03%) than blood based specimens (99.68%), specificity was similar (99.74% oral vs. 99.91% blood). 35 As part of the testing workflow, a specimen is collected via a cheek swab, and results are available before the visit's completion. The resident would then document the results in the patient's chart and inform the patient of the results, along with information and education on PrEP. If a positive result arises, a warm handoff occurs, during which the patient is escorted to the HIV clinic for confirmatory testing and further education ( Fig. 3 ).

To help dental residents effectively implement the proposed workflow, they were provided with trainings, presentations, tip sheets, and videos that offered detailed, step-by-step instructions. For patient interactions, infectious diseases physicians coached the residents, conducted role-playing scenarios, and educated them on how to offer tests, communicate results, and perform warm handoffs. For EHR instructions, realistic training patients were created in a simulation environment so residents could see the CDSS fire and practice the proposed workflow, including ordering tests and documenting results. Finally, a patient navigator (research assistant) who was well-versed in the workflow and sensitive communication methods assisted residents during the initial go-live.

### Assessing the Objectives

The period of when the CDSS was functional and the associated workflow was fully implemented was from September 2022 to June 2023. We planned to assess the effectiveness of the CDSS and workflow in phases:

- Phase 1: a ramp-up period when the study team was available to smooth out issues, answer questions, and enforce workflow integrity (8 weeks).
- Phase 2: a period when the patient navigator was available to assist the residents (12 weeks).
- Phase 3: a period when the patient navigator was no longer available (12 weeks).

While reporting on CDSS firing rates and HIV testing rates by residents helped assess uptake, staying in constant communication with the residents and faculty of the AEGD clinic was critical. The provider buy-in was vital in ensuring smooth execution. Periodic Q&A sessions were held to both help residents and obtain qualitative feedback on the CDSS and workflow.

## Results

Once the CDSS was activated, during Phase 1 (ramp-up period) of implementation, of the 1,613 dental residents' patient visits, 956 (or 59%) prompted the CDSS to alert the provider about the potential need for HIV testing, with a testing rate of 3.1% ( *n* = 30; Table 2 ). This phase was characterized by dental providers acclimating to the new workflow, fine-tuning their time management strategies, gaining confidence in discussing the sensitive topic of HIV testing with patients, and familiarizing themselves with inventory and materials.

**Table 2:** This table details the clinical decision support system data and testing effectiveness in each of the three phases of the implementation

Time period	Ramp up (8 wk)	With navigator (12 wk)	Without navigator (12 wk)
Visits	1,613	2,231	2,595
CDSS alerts	956	893	838
% of CDSS alerts/visits	59%	40%	32%
HIV tests	30	113	96
% of HIV tests/CDSS alerts	3.1%	12.7%	11.5%

Abbreviation: CDSS, clinical decision support system.

During Phase 2, introduction of a patient navigator whose duties included facilitating interactions with patients on the importance of HIV testing and preventions modalities like PrEP had a remarkable impact, leading to a significant increase in HIV testing rates, reaching 12.7% ( *n* = 113; Table 2 ). The presence of the patient navigator played a pivotal role in facilitating and enhancing the testing process. What is particularly encouraging is even after the patient navigator's support was no longer available, the HIV testing rate was 11.5% ( *n* = 96; Table 2 ).

Throughout Phases 2 and 3, the percentage of times the CDSS fired decreased to 40% (893 alerts/2,231 visits) and 32% (838 alerts/2,595 visits), respectively ( Table 2 ). Once the CDSS fired, if its suggestion for HIV testing is not accepted, there is a delay in subsequent firing as follows: 1 day for patient barriers, 7 days for visit barriers, and 6 months for patient refused. Since a significant number of patients return to clinics for multiple appointments, the proportion of CDSS alerts decreased. Overall, 18% of the CDSS alert declinations were due to patient barriers, 52% were due to visit barriers, and 30% were due to patient refusal ( Table 3 ).

**Table 3:** This table details the reasons that dental residents noted for declining the clinical decision support system when it fired an alert

Reasons for declination	Count	% Reason/Total
Patient barriers	387	18
Visit barriers	1,153	52
Patient refused	655	30
Total declinations	2,195	100

## Discussion

The strategy to EHE is to increase testing and provide patients with information regarding PrEP. With an initial 59% CDSS alert rate in our AEGD residency program, this validates the premise for expanding HIV testing. Normalizing HIV POC testing in the dental ambulatory care setting is a key aspect of this program and follows the guidelines recommended by the American Dental Association, CDC, and NYSDOH for HIV testing. 1 36 37

Even without the patient navigator in Phase 3, we observed that HIV testing maintained a consistent level of performance, underscoring the sustained effectiveness of the system in promoting and facilitating testing even in the absence of additional assistance. Our testing rate of approximately 12% is encouraging since a National Hospital Ambulatory Medical Care Survey sampling emergency department visit in the United States in 2018 revealed a testing rate of 1.05%. 38 Additionally, in a small study that did not utilize CDSS's, but instead offered HIV testing based on patient responses to a questionnaire, a testing rate of 8.2% (21/256) was observed. 18

HIV testing was a new process for residents and the supervising faculty, and a few concerns were identified early on; however, through collaborative efforts, we made several modifications, improvements, and iterations in workflows, policies, and the CDSS. For example, a dental assistant lacking the required training would open the patient chart, potentially dismissing the CDSS. Modifying the triggers based on provider role, subsequently prevented this occurrence as it was restricted to alert only residents. With a more efficient protocol, we would expect improved testing rates in further implementations of this CDSS and workflow at other partner dental sites. We identified several limitations of this approach, including patient reluctance to discuss HIV testing in a dental setting, which may lead to lower acceptance rates, as well as challenges in integrating testing into existing workflows, potentially disrupting routine operations and requiring additional time and resources. This was evidenced by the relatively higher percentages of CDSS declinations due to patient refusals and visit barriers, respectively.

Although we preemptively attempted to mitigate anticipated barriers such as resource/time constraints and knowledge gaps, several additional barriers were identified through over-the-shoulder observations, resident Q&A sessions, faculty feedback, and patient navigator input. These included language barriers, availability and location of test kits, adherence to the manufacturer's testing instructions, and faculty supervision. Remediating such barriers would alleviate concerns in the future as well. Finally, promoting HIV testing in dental setting, providing extra training, and coaching residents would further enhance the overall experience.

In reflecting on the aforementioned lessons learned, we realized the complexity involved with introducing HIV testing in the dental setting. These ranged from technical expertise to staffing resources and comprehensive training. Resolving barriers and finalizing CDSS and workflow modifications during this study helped us develop a more detailed and thorough template for future implementations. This is especially important since a new cohort of dental residents matriculate every year; therefore, this detailed documented protocol will become even more valuable.

## Conclusion

An integrated EHR like Epic allows dental providers to access medical records, including diagnoses and key laboratory values, which can help determine the need for guideline-supported HIV testing. EHR data and CDSS tools can effectively facilitate HIV testing by dental providers and contribute to national HIV EHE initiatives. Although our study was limited to a residency program, the dental setting provides an opportunity to expand testing to patients who would otherwise not receive this service.

## Clinical Relevance Statement

The implementation of HIV testing in the dental ambulatory care setting is significant in that this protocol demonstrates the feasibility in expanding the workforce within the health care ecosystem. As primary care networks continue to expand, seizing upon integrated electronic health records and collaboration among health care providers unveils a promising avenue for elevating patient care and health outcomes. Embedding clinical decision support tools into workflows not only facilitates the identification of standards of care but also enables the implementation of public health initiatives within multidisciplinary health care organizations, thus elevating health care standards.

## Multiple Choice Questions

1. Which of the below were incorporated as part of the criteria for identifying patients that would benefit from HIV screening?
  - Significant history of HIV testing
  - Positive herpes results
  - Positive chlamydia results
  - Ages under 18 years
  **Correct Answer:** The correct answer is option c. The overarching logic was either “no history of HIV testing” or a combination of a “negative HIV test” plus either a “positive gonorrhea/chlamydia/syphilis test,” as long as the patient is “18 years old or older.”
2. Which of the below was a step involved in developing the CDS?
  - Identifying qualifying diagnoses codes
  - Utilizing artificial general intelligence
  - Establishing positive and negative laboratory value criteria
  - Disregarding laboratory values in the decision-making process
  **Correct Answer:** The correct answer is option c. The main steps involved identifying qualifying LOINC codes, establishing the correct criteria for interpreting positive and negative laboratory values, and ultimately piecing everything together via rule-based programming.
